# Increased expression of the interleukin-1 receptor-associated kinase (IRAK)-1 is associated with adipose tissue inflammatory state in obesity

**DOI:** 10.1186/s13098-015-0067-7

**Published:** 2015-08-27

**Authors:** Rasheed Ahmad, Puthiyaveetil Kochumon Shihab, Reeby Thomas, Munera Alghanim, Amal Hasan, Sardar Sindhu, Kazem Behbehani

**Affiliations:** Laboratory of Immunology & Innovative Cell Therapy, Dasman Diabetes Institute (DDI), P.O. Box 1180, Dasman, 15462 Kuwait City, Kuwait

**Keywords:** Interleukin-1 receptor-associated kinase, Adapter protein, Adipose tissue, Obesity, Type-2 diabetes, Metabolic inflammation, Meta-inflammation, TLR/IL-1R/MyD88 pathway

## Abstract

**Background:**

The emerging role of TLR2/4 as immuno-metabolic receptors points to key involvement of TLR/IL-1R/MyD88 pathway in obesity/type-2 diabetes (T2D). IL1R-associated kinase (IRAK)-1 is a critical adapter protein (serine/threonine kinase) of this signaling pathway. The changes in adipose tissue expression of IRAK-1 in obesity/T2D remain unclear. We determined modulations in IRAK-1 gene/protein expression in the subcutaneous adipose tissues from lean, overweight and obese individuals with or without T2D.

**Methods:**

A total of 49 non-diabetic (22 obese, 19 overweight and 8 lean) and 42 T2D (31 obese, 9 overweight and 2 lean) adipose tissue samples were obtained by abdominal subcutaneous fat pad biopsy and IRAK-1 expression was determined using real-time RT-PCR, immunohistochemistry, and confocal microscopy. IRAK-1 mRNA expression was compared with adipose tissue proinflammatory mediators (TNF-α, IL-6, IL-18), macrophage markers (CD68, CD11c, CD163), and plasma markers (CCL-5, C-reactive protein, adiponectin, and triglycerides). The data were analyzed using *t* test, Pearson’s correlation, and multiple stepwise linear regression test.

**Results:**

In non-diabetics, IRAK-1 gene expression was elevated in obese (*P* = 0.01) and overweight (*P* = 0.04) as compared with lean individuals and this increase correlated with body mass index (r = 0.45; *P* = 0.001) and fat percentage (r = 0.36; *P* = 0.01). In diabetics, IRAK-1 mRNA expression was also higher in obese as compared with lean subjects (*P* = 0.012). As also shown by immunohistochemistry/confocal microscopy in non-diabetics and by immunohistochemistry in diabetics, IRAK-1 protein expression was higher in obese than overweight and lean adipose tissues. IRAK-1 gene expression correlated positively/significantly with mRNAs of TNF-α (r = 0.46; *P* = 0.0008), IL-6 (r = 0.30; *P* = 0.03) and IL-18 (r = 0.31; *P* = 0.028) in non-diabetics; and only with TNF-α (r = 0.32; *P* = 0.03) in diabetics. IRAK-1 expression also correlated positively/significantly with CD68 (r = 0.32; *P* = 0.02), CD11c (r = 0.30; *P* = 0.03), and CD163 (r = 0.43; *P* = 0.001) in non-diabetics; and only with CD163 (r = 0.34; *P* = 0.02) in diabetics. IRAK-1 mRNA levels also correlated with plasma markers including CCL-5 (r = 0.39; *P* = 0.02), C-reactive protein (r = 0.48; *P* = 0.005), adiponectin (r = −0.36; *P* = 0.04), and triglycerides (r = 0.40; *P* = 0.02) in non-diabetics; and only with triglycerides (r = −0.36; *P* = 0.04) in diabetics. IRAK-1 expression related with TLR2 (r = 0.39; *P* = 0.007) and MyD88 (r = 0.36; *P* = 0.01) in non-diabetics; and MyD88 (r = 0.52; *P* = 0.0003) in diabetics.

**Conclusions:**

The elevated IRAK-1 expression in obese adipose tissue showed consensus with local/circulatory inflammatory signatures and represented as a tissue marker for metabolic inflammation. The data have clinical significance as interventions causing IRAK-1 suppression may alleviate meta-inflammation in obesity/T2D.

**Electronic supplementary material:**

The online version of this article (doi:10.1186/s13098-015-0067-7) contains supplementary material, which is available to authorized users.

## Background

Modern lifestyle has led to the reduced physical activity and increased consumption of energy-rich foods which are the root cause of the substantial increase in obesity worldwide. Obesity is known to induce a state of chronic low-grade systemic inflammation in both humans and rodents in which the expanded adipose tissue functions as an active endocrine organ and secretes adipokines including proinflammatory cytokines/chemokines, hormones, and other mediators [1–4]. Toll-like receptors (TLRs) are the membrane-spanning non-catalytic innate immune receptors that recognize pathogen-associated molecular patterns (PAMPs) and induce inflammatory responses. Free fatty acids, in addition to PAMPs, also act as TLR agonists [5] which points to the emerging role of TLRs as immunometabolic receptors in obesity. Interleukin (IL)-1 receptor-associated kinases (IRAKs) are the death domain containing serine/threonine kinases and adapter proteins that play a crucial role in signaling cascades of IL-1 family receptors and TLRs. TLR signaling is initiated in the intracellular toll/IL-1 receptor (TIR) domain and is classified into myeloid differentiation factor 88 (MyD88)-dependent or MyD88-independent (TRIF-dependent) pathways [6, 7]. The MyD88-dependent signaling is induced by TLR dimerization after the TLR engagement with cognate ligands, and results in the recruitment of MyD88 and IRAKs to the TIR domain [8, 9]. IRAK-1 is activated after phosphorylation by IRAK-4 and associates with TNF receptor-associated factor (TRAF)-6. The IRAK-1/TRAF-6 complex further recruits TGFβ-activated kinase (TAK)-1 and TAB-1/2 adapter proteins to form a macromolecular complex [10]. Hyperphosphorylated IRAK-1 dissociates from the signaling complex and TAK-1 activates the inhibitor of nuclear factor kappa (NF-κ)-B kinase alpha/beta (IKKα/β) to result in IκBα phosphorylation, ubiquitination, degradation, and nuclear translocation of p65 NF-κB complexes [11]. Simultaneously, TAK-1 phosphorylates members of the mitogen-activated protein kinase (MAPK) kinase i.e. MKK family including MKK4, MKK3, or MKK6 which, in turn, activate c-Jun N-terminal kinase (JNK) and p38 MAPK.

The immunometabolic role of TLR2 and TLR4 makes these receptors and their associated pathways critical for identifying targets to treat metabolic conditions such as obesity-induced inflammation and type-2 diabetes (T2D). Obesity leads to the enhanced expression of TLR2 and TLR4 on the peripheral blood mononuclear cells which has significant implications with regard to inflammation [12]. However, it remains unclear how the expression of IRAK-1 adapter protein, which is a critical component of the TLR/IL-1R/MyD88 pathway, is modulated in obesity or T2D. White adipose tissue is critical with regard to obesity and its metabolic complications, including T2D, as it is the primary site for energy storage and is also an active endocrine organ while the brown adipose tissue is related with energy expenditure and regulates non-shivering thermogenesis [13]. We hypothesized that obesity was an inducer or a positive modulator of IRAK-1 expression in the human white adipose tissue. Herein, we present the data from both non-diabetic and diabetic individuals showing higher IRAK-1 gene and protein expression in the subcutaneous adipose tissues of obese or overweight subjects as compared with lean counterparts. The correlations of IRAK-1 gene expression were assessed against clinical markers of obesity, local proinflammatory mediators, monocyte/macrophage markers, systemic inflammatory markers, and more importantly, against upstream signaling components of the TLR/IL-1R/MyD88 pathway.

## Patients and methods

### Study population

A total of 49 non-diabetic (25 male and 24 female, aged 26–71 years) and 42 diabetic (24 male and 18 female, aged 23–72 years) individuals were recruited in the study. The participants were classified as lean, overweight, and obese based on BMI index. The non-diabetic group comprised of 8 lean (BMI = 23.035 ± 2.326 kg/m^2^; 2 male/6 female), 19 overweight (BMI = 28.31 ± 1.081 kg/m^2^; 12 male/7 female), and 22 obese (BMI = 34.383 ± 2.784 kg/m^2^; 11 male/11 female) individuals. The diabetic group comprised of 2 lean (BMI = 25.473 ± 0.338 kg/m^2^; 1 male/1 female), 9 overweight (BMI = 28.130 ± 1.015 kg/m^2^; 5 male/4 female), and 31 obese (BMI = 33.759 ± 2.561 kg/m^2^; 18 male/13 female) individuals. Different morbid conditions in non-diabetic group included hypertension (5), hyperlipidemia (2), coronary artery disease (1), allergy (1), and asthma (1). In diabetic group, comorbidities included hypertension (17), hyperlipidemia (5), coronary artery disease (2), allergy (2), and asthma (3). The clinico-demographic data of the participants are summarized in Table 1. All participants gave written informed consent and the study was approved by the ethics committee of Dasman Diabetes Institute, Kuwait.

**Table 1:** Patients’ characteristics and clinical data

Parameter	Non-diabetic			Diabetic		
	Lean	Overweight	Obese	Lean	Overweight	Obese
Total number (N)	8	19	22	2	9	31
Male (N)	2	12	11	1	5	18
Female (N)	6	7	11	1	4	13
Age (years)	28–53	29–71	26–66	48–58	45–59	23–72
Body mass index (kg/m^2^)	23.035 ± 2.326	28.31 ± 1.081	34.383 ± 2.784	25.473 ± 0.338	28.130 ± 1.015	33.759 ± 2.561
Fat percentage (fat %)	29.757 ± 6.196	32.647 ± 4.998	39.011 ± 4.350	32.100 ± 6.223	33.050 ± 5.379	37.333 ± 5.035
Glucose (mmol/l)	5.096 ± 0.639	5.602 ± 1.559	5.424 ± 0.799	5.800 ± 0.424	8.578 ± 1.770	8.640 ± 2.930
Cholesterol (mmol/l)	5.313 ± 1.254	4.899 ± 0.773	5.233 ± 1.073	5.400 ± 2.546	4.816 ± 1.681	5.065 ± 1.236
High-density lipoprotein (mmol/l)	1.791 ± 0.477	1.219 ± 0.225	1.115 ± 0.235	1.135 ± 0.148	1.147 ± 0.411	1.129 ± 0.288
Low-density lipoprotein (mmol/l)	3.250 ± 1.046	3.147 ± 0.699	3.427 ± 0.942	3.700 ± 2.121	2.767 ± 1.391	3.013 ± 1.106
Triglycerides (mmol/l)	0.566 ± 0.216	1.215 ± 0.644	1.465 ± 0.821	1.245 ± 1.252	1.972 ± 0.880	1.913 ± 1.495
HbA1c (%)	5.688 ± 0.464	5.917 ± 1.65	5.863 ± 0.497	6.000 ± 0.283	7.544 ± 1.673	8.235 ± 1.506
Hypertension (N)	0	2	3	0	3	14
Hyperlipidemia (N)	0	0	2	0	2	3
Coronary artery disease (N)	0	0	1	0	1	1
Allergy (N)	0	0	1	0	0	2
Asthma (N)	0	1	0	0	0	3
Therapy		Nadolol, Lipitor, Aspirin	Concor, Lipitor, Aspirin, Diovan	Metfornin, Aspirin	Glucophage, Lipitor, Lantus, Zocor, Diovan, Metformin, Aspirin	NovoRapid, Lantus, Insulin, Aspirin, Concor, Zocor, Mixtard, Glucophage, Metformin, Capoten

### Anthropometric and physio-clinical measurements

Anthropometric and physical measurements included body weight, height, waist circumference as well as systolic and diastolic blood pressure. Height and weight were measured with barefoot participants wearing light indoor clothing using calibrated portable electronic weighing scales and portable inflexible height measuring bars; the waist circumference at the highest point of the iliac crest and the mid-axillary line was measured using constant tension tape at the end of a normal expiration with arms relaxed at the sides. The whole body composition including body fat percentage (fat %), soft lean mass and total body water were measured using IOI 353 Body Composition Analyzer (Jawon Medical, South Korea). Blood pressure was measured by using Omron HEM-907XL digital automatic sphygmomanometer (Omron Healthcare Inc. IL, USA). An average of the 3 blood pressure readings, with 5–10 min rest between each, was obtained. BMI was calculated using the standard BMI formula i.e. body weight (kg)/height (m^2^).

Regarding clinical laboratory measurements, peripheral blood was collected by phlebotomist through venipuncture from overnight-fasted (minimum 10 h) individuals and the samples were analyzed for fasting glucose, glycated hemoglobin (HbA1c), fasting insulin, and lipid profile. Glucose and lipid profiles were measured using Siemens dimension RXL chemistry analyzer (Diamond Diagnostics, Holliston, MA, USA). Glycated hemoglobin (HbA1c) was measured by using Variant™ device (BioRad, Hercules, CA, USA). To determine adipokine levels, blood in EDTA vacutainer tubes was centrifuged at 1200×*g* for 10 min and plasma was collected, aliquoted and stored at −80 °C until use. Plasma CCL5 (Intra-assay CV % = 3.6; Inter-assay CV % = 10.3) and adiponectin (Intra-assay CV % = 3.7; Inter-assay CV % = 8.5) were assessed using immunobead assays (Luminex, Austin, TX, USA) and high sensitivity C-reactive protein (hsCRP) levels were measured using ELISA kit (Intra-assay CV % = 4.1; Inter-assay CV % = 6.3) (Biovendor, USA). Plasma triglycerides were measured using commercial kit (Intra-assay CV % = 0.93; Inter-assay CV % = 3.05) (Chema Diagnostica, Monsano, Italy). All these assays were carried out following instructions as recommended by the manufacturers.

### Collection of subcutaneous adipose tissue samples

Human adipose tissue samples (~0.5 g) were collected via abdominal subcutaneous fat pad biopsy lateral to the umbilicus using standard surgical method. Briefly, the periumbilical area was sterilized by alcohol swabbing and then locally anesthetized using 2 % lidocaine (2 ml). Through a small superficial skin incision (0.5 cm), fat tissue was collected. After removal, the biopsy tissue was further incised into smaller pieces, rinsed in cold phosphate buffered saline (PBS), fixed in 4 % paraformaldehyde for 24 h and then embedded in paraffin for further use. At the same time, freshly collected adipose tissue samples (~50–100 mg) were preserved in RNAlater or embedded in optimal cutting temperature (OCT) medium and stored at −80 °C until use.

### Real-time reverse-transcription polymerase chain reaction (RT-PCR)

Total cellular RNA was purified using RNeasy kit (Qiagen, Valencia, CA, USA) as per manufacturer’s instructions. Briefly, the adipose tissue samples in RNAlater or OCT-embedded were thawed and homogenized in Qiazol lysis solution (Qiagen, Valencia, CA, USA) using TissueRuptor (Qiagen, Hildon, Germany) at 33,000 rpm for 40 s. The homogenate was treated with chloroform and separated into aqueous and organic phases by centrifugation at 12,000×*g* for 15 min at 4 °C. The upper aqueous RNA phase was collected, 70 % ethanol was added, and the sample was applied to an RNeasy spin column to allow total RNA binding with the membrane and to wash out phenol and other contaminants. High-quality RNA was then eluted in RNase-free water. The quantity of the isolated RNA was determined using Epoch™ Spectrophotometer System (BioTek, Winooski, USA) and the quality was assessed by formaldehyde-agarose gel electrophoresis. The RNA samples (1 μg each) were reverse transcribed to yield cDNA using random hexamer primers and TaqMan reverse transcription reagents (High Capacity cDNA Reverse Transcription kit; Applied Biosystems, CA, USA).

For real-time RT-PCR, cDNA (50 ng) was amplified using TaqMan^®^ Gene Expression MasterMix (Applied Biosystems, CA, USA) and gene-specific 20× TaqMan Gene Expression Assays as follows: (IRAK-1) Hs01018347_m1; (TNF-α) Hs01113624_g1; (IL-6) Hs00985639_m1; (IL-18) Hs01038788_m1; (CD68) Hs02836816_g1; (CD11c) Hs00174217_m1; (CD163) Hs00174705_m1; (TLR2) Hs01872448_s1; (TLR4) Hs00152939_m1; (MyD88) Hs01573837_g1; and (GAPDH) Hs03929097_g1 (Applied Biosystems, CA, USA) containing forward and reverse primers and a target-specific TaqMan^®^ minor groove binder (MGB) probe labeled with 6-fluorescein amidite (FAM) dye at the 5′ end and non-fluorescent quencher (NFQ)-MGB at the 3′ end of the probe, for 40 cycles of PCR reaction using a 7500 Fast Real-Time PCR System (Applied Biosystems, CA, USA). Each cycle consisted of denaturation for 15 s at 95 °C, annealing/extension for 1 min at 60 °C which started after uracil DNA glycosylase (UDG) activation (50 °C for 2 min) and AmpliTaq Gold enzyme activation (95 °C for 10 min). The amplified glyceraldehyde 3-phosphate dehydrogenase (GAPDH) expression was used as internal control to normalize the differences in individual samples and gene expression level of IRAK-1 relative to controls (lean adipose tissue) was calculated using 2^−ΔΔCt^ method. Relative mRNA expression was measured as fold expression over average of control gene expression. The expression level in control samples was assumed as 1 and data were presented as mean ± SEM values.

### Immunohistochemistry

Paraffin-embedded sections (4 µm thick) of subcutaneous adipose tissue were deparaffinized in xylene and rehydrated through descending grades of ethanol (100, 95, and 75 %) to water. Antigen retrieval was performed by placing slides in target retrieval solution (pH 6.0; Dako, Glostrup, Denmark) in the pressure cooker boiling for 8 min and cooling for 15 min. After washing in PBS, endogenous peroxidase activity was blocked with 3 % H_2_O_2_ for 30 min and non-specific antibody binding was blocked with 5 % nonfat milk for 1 h followed by 1 % bovine serum albumin solution for 1 h. The slides were incubated at room temperature overnight with primary antibody (1:100 dilution of rabbit polyclonal anti-IRAK antibody; Abcam^®^ ab62700). After washing with PBS (0.5 % Tween), slides were incubated for 1 h with secondary antibody (goat anti-rabbit conjugated with horse radish peroxidase (HRP) polymer chain; EnVision™ Kit from Dako, Glostrup, Denmark) and color was developed using 3,3ʹ-diaminobenzidine (DAB) chromogen substrate. Specimens were washed in running tap water, lightly counterstained with Harris hematoxylin, dehydrated through ascending grades of ethanol (75, 95, and 100 %), cleared in xylene, and finally mounted in dibutyl phthalate xylene (DPX).

For analysis, digital photomicrographs of the entire adipose tissue sections (20×; Olympus BX51 Microscope, Japan) were used to quantify the immunohistochemical staining in three different regions to assess the regional heterogeneity in tissue samples and the regions were outlined using Aperio ImageScope software (Aperio Vista, CA, USA). The Aperio-positive pixel count algorithm (version 9) was used to quantify the intensity of specific staining in the region. The number of positive pixels was normalized to the number of total (positive and negative) pixels to account for variations in the size of the region sampled. Color and intensity thresholds were established to detect the immunostaining as positive and background staining as negative pixels. Once the conditions were established, all slides were analyzed using the same parameters. The resulting color markup of the analysis was confirmed for each slide.

### Confocal microscopy

Formalin-fixed and paraffin-embedded sections (8 μm) of subcutaneous adipose tissue were processed for immunofluorescent labeling using similar protocol as described for immunohistochemistry. After antigen retrieval and blocking, the samples were incubated overnight at room temperature with primary antibody (1:100 dilution of rabbit polyclonal anti-IRAK antibody) (abcam^®^ ab62700). After washing twice with PBS-0.05 % Tween, slides were incubated for 1 h with secondary antibody (1:1000 dilution of goat anti-rabbit antibody conjugated with Alexa Fluor^®^ 488; Abcam^®^ ab150077) and washed at least thrice in PBS. The samples were counterstained with 4′,6-diamidino-2-phenylindole (DAPI) (Vectashield, Vectorlab, -H1500) and mounted with cover slips. For image processing and analysis, confocal images of the adipose tissue were collected on inverted Zeiss LSM710 Spectral confocal microscope (Carl Zeiss, Gottingen, Germany) using EC Plan-Neofluar 40×/1.30 oil DIC M27 objective lens. Samples were excited using a 488 nm diode-pumped solid-state laser and the 405 nm line of an argon ion laser. After laser excitation of the samples, optimized emission detection bandwidths were configured by using Zeiss Zen 2010 control software.

### Statistical analysis

The data obtained were expressed as mean ± SEM values. The group means of IRAK-1 gene expression data, after checking for normal distribution, were compared using unpaired *t*-test and the linear dependence between two variables was assessed by Pearson’s correlation coefficient ‘r’ values. Mann–Whitney *U* test was used to compare means of IRAK-1 protein expression data regarding lean, overweight and obese adipose tissue samples. SPSS Statistics 20 software (IBM Inc. USA) was used to perform multiple stepwise linear regression analysis in order to determine which variables were independently associated as predictors with IRAK-1. GraphPad Prism software (version 6.05; San Diego, CA, USA) was used for statistical analysis and graphical representation of the data. All *P*-values ≤0.05 were considered statistically significant.

## Results

### Adipose tissue IRAK-1 expression is significantly elevated in obese individuals with or without T2D

**Fig. 1:**
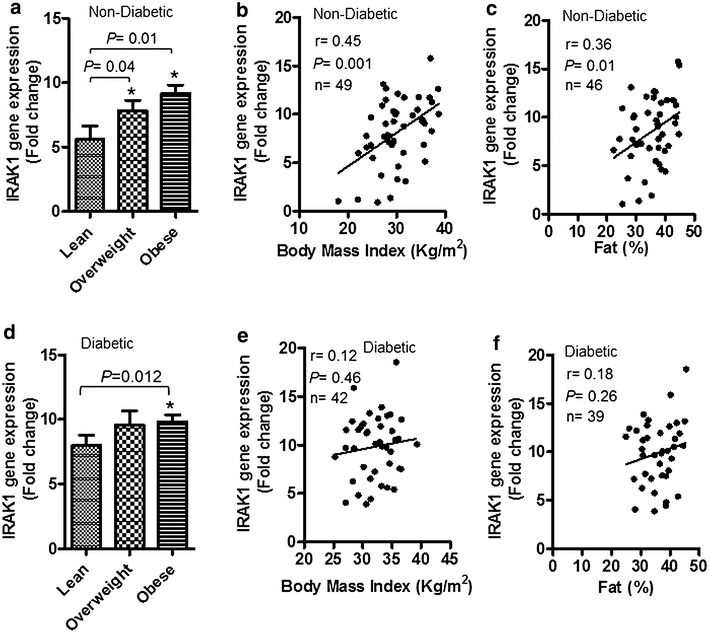
Adipose tissue IRAK-1 gene expression is significantly upregulated in obese non-diabetic/diabetic individuals. IL-1 receptor-associated kinase (IRAK)-1 gene expression in the subcutaneous adipose tissue biopsy samples collected from 49 non-diabetic (22 obese, 19 overweight, and 8 lean) and 42 type-2 diabetic (T2D) (31 obese, 9 overweight, and 2 lean) individuals was determined by real-time RT-PCR as described in “Patients and methods”. **a** IRAK-1 mRNA expression in non-diabetics was found to be significantly elevated in the adipose tissues of obese (*P* = 0.01) or overweight individuals (*P* = 0.04) as compared with lean counterparts. The increased IRAK-1 gene expression correlated positively with **b** body mass index (BMI) (r = 0.45; *P* = 0.001) and **c** body fat percentage (fat %) (r = 0.36; *P* = 0.01). **d** IRAK-1 mRNA expression in T2D patients was also found to be significantly elevated in the adipose tissues of obese (*P* = 0.012) as compared with lean subjects. However, IRAK-1 gene expression did not correlate with **e** BMI (r = 0.12; *P* = 0.46) and **f** fat % (r = 0.18; *P* = 0.26). Due to missing fat % data in non-diabetics and diabetics, 3 each, respective individuals had to be eliminated from IRAK-1 correlation analysis with fat %

IRAK-1 is a key mediator of the TLR/IL-1R signaling cascade. We asked whether obesity modulated the expression of IRAK-1 in the adipose tissue. To this end, we found that in non-diabetic individuals, IRAK-1 gene expression in the adipose tissue was significantly elevated in obese (*P* = 0.01) and overweight (*P* = 0.04) individuals as compared with lean subjects (Fig. 1a). This increase in the IRAK-1 gene expression correlated positively with clinical indicators of obesity such as BMI (r = 0.45; *P* = 0.001) (Fig. 1b) and fat % (r = 0.36; *P* = 0.01) (Fig. 1c). In diabetic patients, IRAK-1 mRNA expression in the adipose tissue was also significantly higher in obese as compared with lean patients (*P* = 0.012) (Fig. 1d); however, IRAK-1 gene expression did not correlate with BMI (r = 0.12; *P* = 0.46) and fat % (r = 0.18; *P* = 0.26) (Fig. 1e, f).

**Fig. 2:**
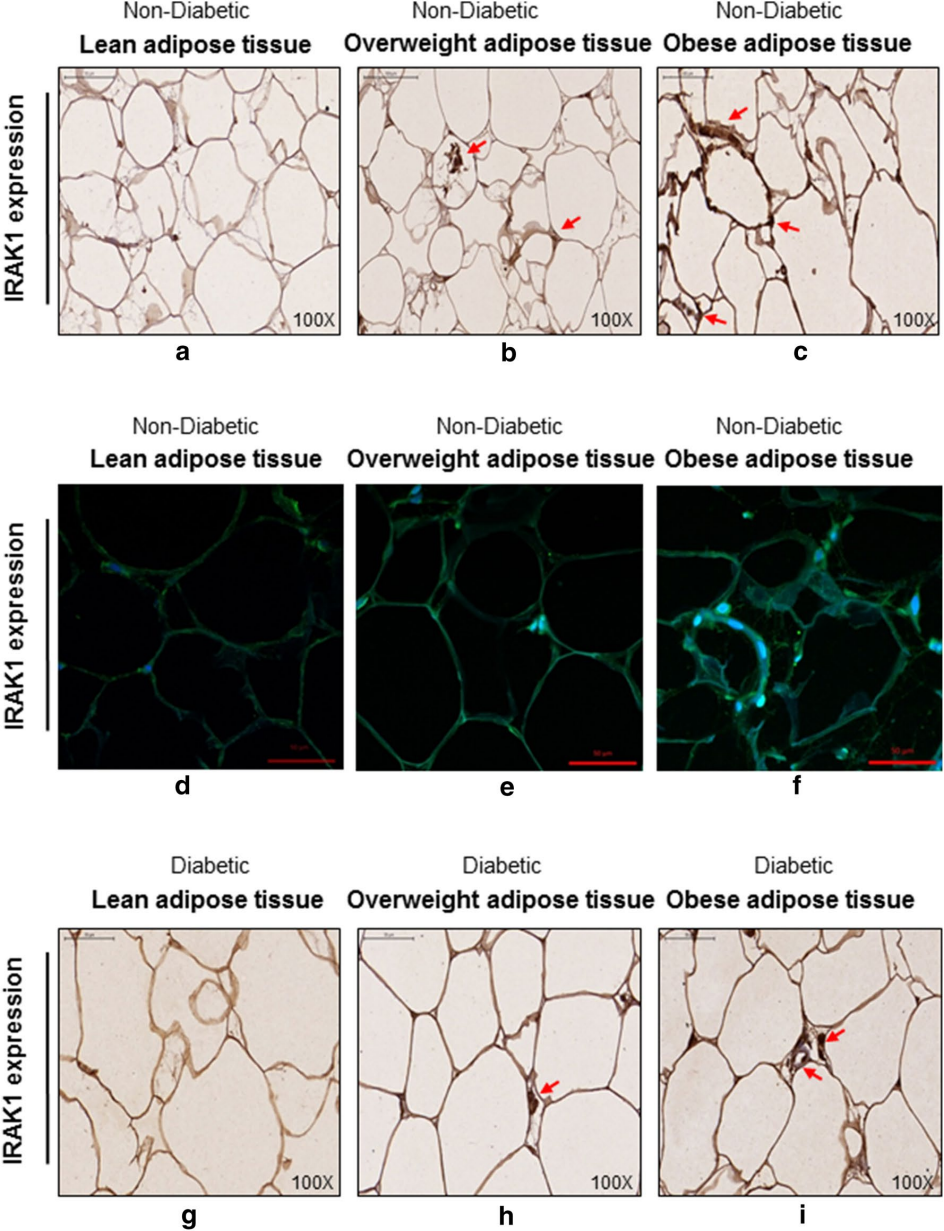
IRAK-1 adipose tissue protein expression is also higher in non-diabetic or diabetic obese individuals. The IRAK-1 protein expression in the subcutaneous adipose tissues of 5 non-diabetic individuals was detected by immunohistochemistry/immunofluorescent staining, and in 3 diabetic individuals by immunohistochemistry as described in “Patients and methods”. The representative photomicrographs show higher IRAK-1 expression (*arrows*) in the adipose tissues of obese non-diabetic individuals using **a**–**c** immunohistochemistry (×100 magnification) and **d**–**f** immunofluorescent staining (×40 magnification). The higher IRAK-1 adipose tissue expression (*arrows*) in obese diabetic individuals is shown (**g**–**i**) by using immunohistochemistry (×100 magnification)

IRAK-1 protein expression in non-diabetics was also found to be enhanced in the adipose tissue of obese and overweight individuals as compared with lean subjects as shown by both immunohistochemistry (Fig. 2a–c) and confocal microscopy (Fig. 2d–f). Comparison of the immunohistochemistry data (5 individuals per group) of specific staining intensity quantification using Aperio-positive pixel count algorithm showed that the IRAK-1 protein expression was significantly higher in obese as compared with lean individuals (*P* = 0.037) (Additional file 1: Figure S1). The tabulated data (Additional file 2: Figure S2) compare the gene and protein expression in this patient cohort. In diabetics, IRAK-1 protein expression in the adipose tissue was also significantly higher in obese and overweight individuals as compared with lean subjects as shown by immunohistochemistry (Fig. 2g–i).

### Increased adipose tissue expression of IRAK-1 mRNA is concordant with local expression of proinflammatory mediators

**Fig. 3:**
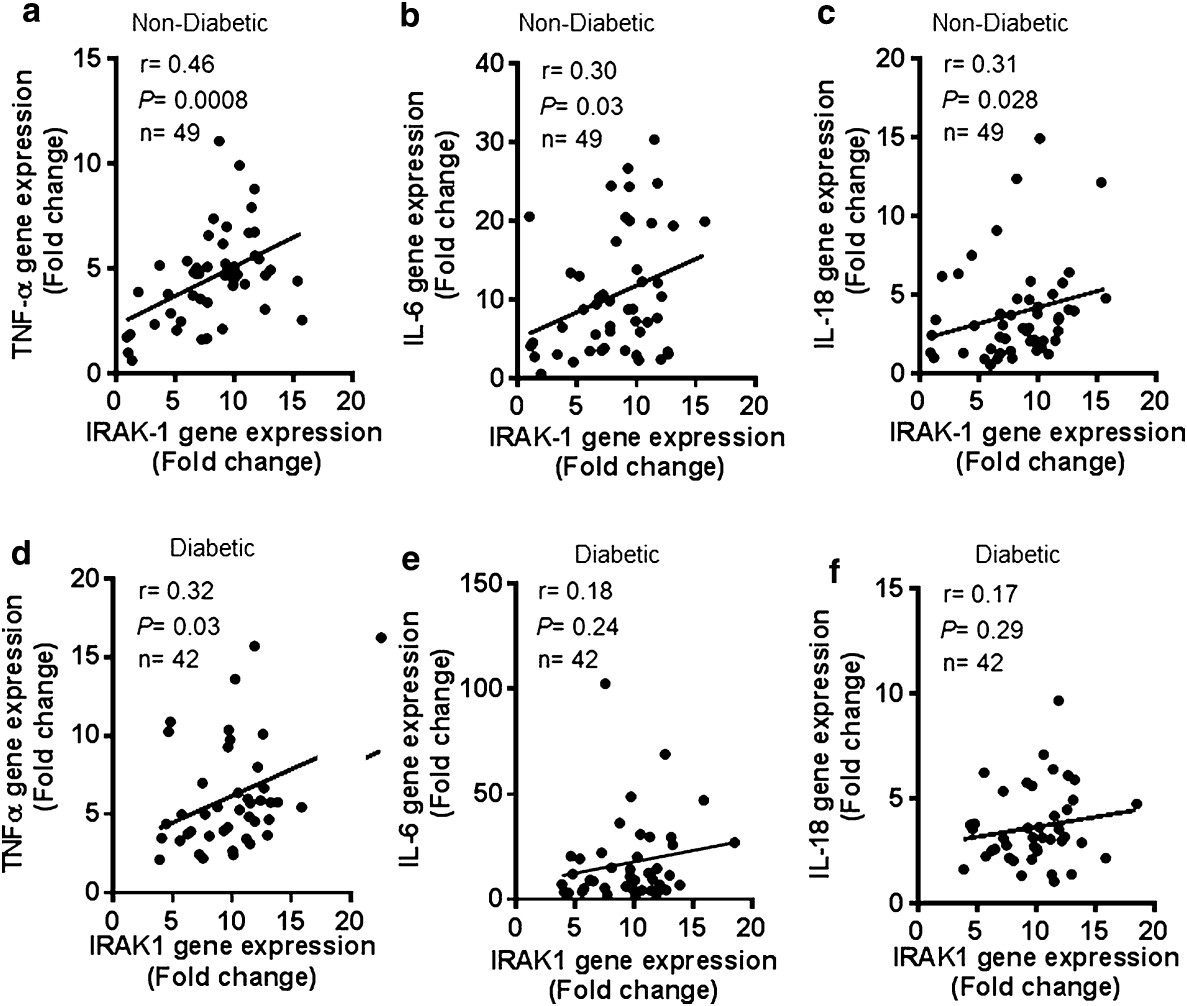
IRAK-1 mRNA expression is concordant with adipose tissue expression of proinflammatory mediators. The adipose tissue gene expression of IRAK-1 and representative proinflammatory mediators such as TNF-α, IL-6, and IL-18 was determined in 49 non-diabetic and 42 diabetic individuals by using real-time RT-PCR as described in “Patients and methods”. The data show that IRAK-1 gene expression in non-diabetic individuals correlated positively/significantly with **a** TNF-α (r = 0.46, *P* = 0.0008); **b** IL-6 (r = 0.30, *P* = 0.03); and **c** IL-18 (r = 0.31, *P* = 0.028). IRAK-1 gene expression in diabetic patients correlated positively/significantly with **d** TNF-α (r = 0.32, *P* = 0.03), while it did not correlate with **e** IL-6 (r = 0.18, *P* = 0.24) and** f** IL-18 (r = 0.17, *P* = 0.29)

Tumor necrosis factor (TNF)-α, IL-6, and IL-18 are the signature proinflammatory cytokines. We next investigated whether the IRAK-1 gene expression in the adipose tissue of non-diabetic or diabetic individuals was concordant with local expression of representative inflammatory mediators or markers. To this effect, we found that IRAK-1 mRNA expression in non-diabetics correlated positively/significantly with the expression of TNF-α (r = 0.46, *P* = 0.0008) (Fig. 3a), IL-6 (r = 0.30, *P* = 0.03) (Fig. 3b), and IL-18 (r = 0.31, *P* = 0.028) (Fig. 3c). Multiple stepwise linear regression analysis showed that TNF-α (*P* < 0.0001) and IL-18 (*P* = 0.025) were independent predictors of the adipose tissue inflammatory marker IRAK-1. However, in diabetic population, the adipose tissue IRAK-1 gene expression correlated significantly only with TNF-α (r = 0.32, *P* = 0.03) (Fig. 3d) and did not correlate with IL-6 (r = 0.18, *P* = 0.24) and IL-18 (r = 0.17, *P* = 0.29) (Fig. 3e, f, respectively).

### Enhanced IRAK-1 gene expression correlates with adipose tissue infiltration by monocytes/macrophages

**Fig. 4:**
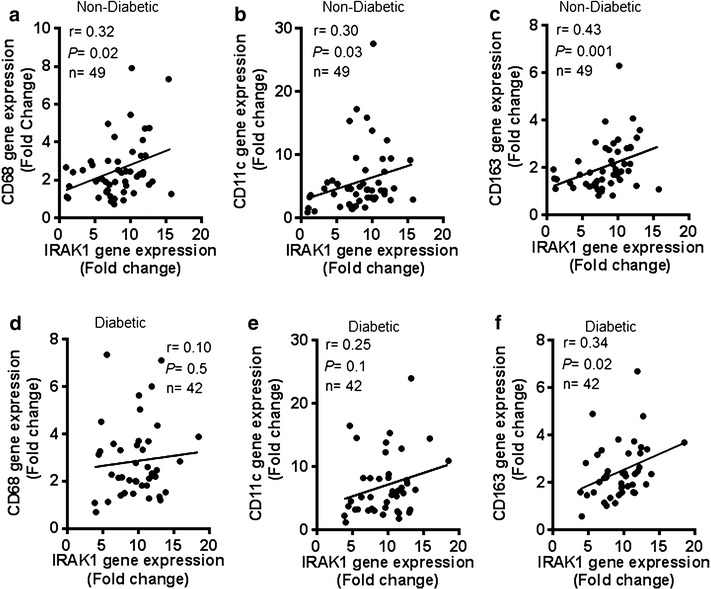
Increased IRAK-1 gene expression correlates with adipose tissue infiltration by monocytes/macrophages. The adipose tissue gene expression of IRAK-1 and selective monocytes/macrophage markers including CD68, CD11c, and CD163 was determined in 49 non-diabetic and 42 diabetic individuals by using real-time RT-PCR. The data show that IRAK-1 gene expression in non-diabetic individuals correlated positively/significantly with **a** CD68 (r = 0.32, *P* = 0.02); **b** CD11c (r = 0.30, *P* = 0.03); and **c** CD163 (r = 0.43, *P* = 0.001). IRAK-1 gene expression in diabetic patients did not associate with **d** CD68 (r = 0.10, *P* = 0.50) and **e** CD11c (r = 0.25, *P* = 0.10); however, it correlated significantly with **f** CD163 expression (r = 0.34, *P* = 0.02)

Macrophages are the key mediators of obesity-associated chronic low-grade metabolic inflammation, also called meta-inflammation. We next asked whether the increased IRAK-1 mRNA expression in the obese adipose tissue of non-diabetic and diabetic individuals was supported by monocyte/macrophage infiltration in this compartment. To this line of investigation, we found that the increased IRAK-1 mRNA expression in non-diabetic obese individuals correlated positively with the expression of CD68 (r = 0.32, *P* = 0.02) (Fig. 4a), CD11c (r = 0.30, *P* = 0.03) (Fig. 4b), and CD163 (r = 0.43, *P* = 0.001) (Fig. 4c). In diabetics, IRAK-1 gene expression did not associate with CD68 (r = 0.10, *P* = 0.50) and CD11c (r = 0.25, *P* = 0.10) (Fig. 4d, e, respectively); but it correlated significantly with CD163 marker (r = 0.34, *P* = 0.02) (Fig. 4f).

### Adipose tissue IRAK-1 mRNA expression correlates with systemic inflammatory or obesity clinical markers

**Fig. 5:**
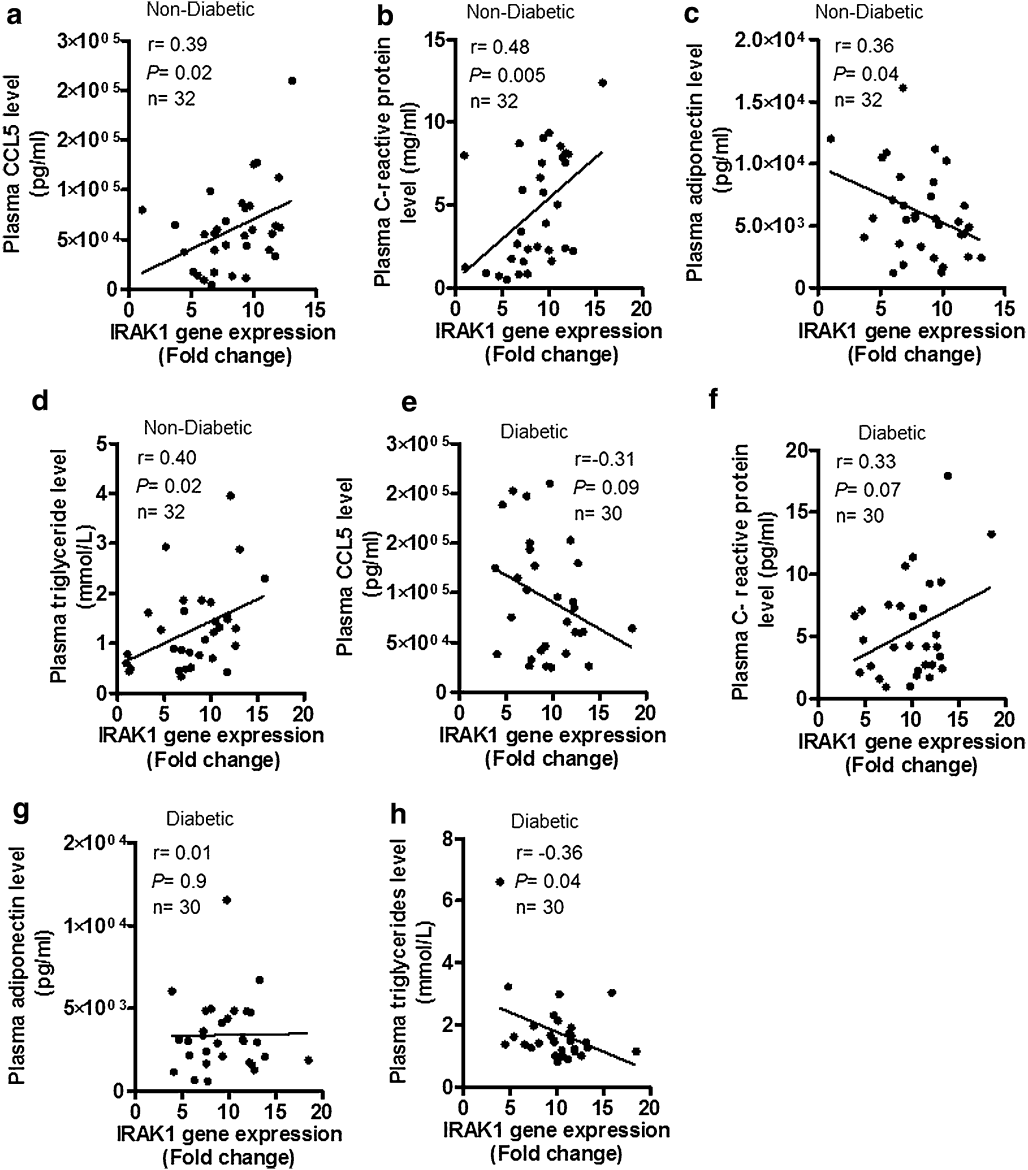
Adipose tissue IRAK-1 mRNA levels correlate with systemic inflammatory or obesity clinical markers. In non-diabetic (49) and diabetic (42) individuals, IRAK-1 gene expression in the adipose tissue was determined using real-time RT-PCR. Plasma levels of representative systemic inflammatory or obesity clinical markers, such as CCL5, C-reactive protein, adiponectin, and triglycerides were determined by using commercial kits and following the manufacturers’ instructions as described in “Patients and methods”. The data show that IRAK-1 mRNA expression in non-diabetic individuals correlated positively/significantly with plasma levels of **a** CCL5 (r = 0.39, *P* = 0.02); **b** C-reactive protein (r = 0.48, *P* = 0.005); **c** adiponectin (r = 0.36, *P* = 0.04); and **d** triglycerides (r = 0.40, *P* = 0.02). The IRAK-1 gene expression in diabetic patients did not associate with plasma levels of **e** CCL5 (r = 0.31, *P* = 0.09); **f** C-reactive protein (r = 0.33, *P* = 0.07); and **g** adiponectin (r = 0.01, *P* = 0.90) but it correlated significantly with **h** triglyceride levels (r = −0.36, *P* = 0.04). Notably, the IRAK-1 correlation data are shown for 32 non-diabetic and 30 diabetic individuals as missing plasma data necessitated exclusion of the relevant individuals from correlation analysis

Plasma CCL-5 (also called regulated on activation, normal T cell expressed and secreted or RANTES), and CRP are typical markers of systemic inflammation while the increased triglyceride levels are clinical indicator of obesity. We next assessed whether the obesity-associated changes in the adipose tissue expression of IRAK-1 mRNA resonated with plasma levels of these markers. To this end, we found that the adipose tissue expression of IRAK-1 mRNA in non-diabetics correlated with plasma levels of CCL-5 (r = 0.39, *P* = 0.02) (Fig. 5a), CRP (r = 0.48, *P* = 0.005) (Fig. 5b), adiponectin (r = −0.36, *P* = 0.04) (Fig. 5c), and triglycerides (r = 0.40, *P* = 0.02) (Fig. 5d). In diabetic patients, IRAK-1 mRNA expression did not associate with CCL-5 (r = −0.31, *P* = 0.09) (Fig. 5e), CRP (r = 0.33, *P* = 0.07) (Fig. 5f), and adiponectin (r = 0.01, *P* = 0.9) (Fig. 5g); however, it correlated significantly with triglyceride levels (r = −0.36, *P* = 0.04) (Fig. 5h).

### IRAK-1 gene expression in the adipose tissue correlates with TLR2 and/or MyD88 gene expression

**Fig. 6:**
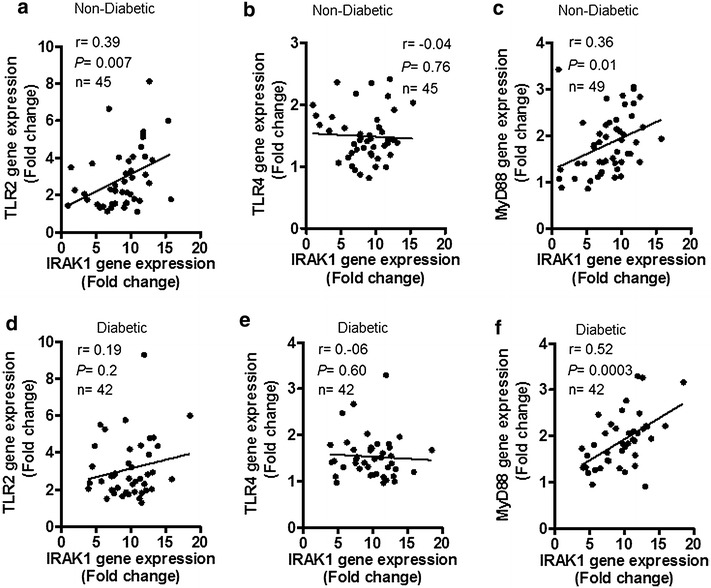
IRAK-1 gene expression in the adipose tissue correlates with TLR2 and/or MyD88 gene expression. The adipose tissue gene expression of IRAK-1 and *upstream* signaling components of the TLR/IL-1R/MyD88 pathway including TLR2, TLR4, and MyD88 was determined in 49 non-diabetic and 42 diabetic individuals by using real-time RT-PCR as described in “Patients and methods”. The data show that IRAK-1 gene expression in non-diabetic individuals correlated positively/significantly with **a** TLR2 (r = 0.39, *P* = 0.007) and **c** MyD88 (r = 0.36, *P* = 0.01) but did not correlate with **b** TLR4 (r = −0.04, *P* = 0.76). IRAK-1 gene expression in diabetic patients did not associate with **d** TLR2 (r = 0.19, *P* = 0.20) and **e** TLR4 (r = −0.06, *P* = 0.60); however, it correlated significantly with **f** MyD88 expression (r = 0.52, *P* = 0.0003). The IRAK-1 correlation with TLR2 and TLR4 is shown for 45 non-diabetic individuals, each, as the missing TLR2/4 data necessitated exclusion of the relevant individuals from correlation analysis

We next asked if the elevated IRAK-1 mRNA expression in the obese adipose tissue was related with signaling molecules located upstream in the TLR/IL-1R/MyD88 cascade. To this effect, we found that in non-diabetic individuals, IRAK-1 gene expression correlated positively/significantly with TLR2 (r = 0.39, *P* = 0.007) and MyD88 expression (r = 0.36, *P* = 0.01) and did not associate with TLR4 (r = −0.04, *P* = 0.76) (Fig. 6a–c). However, in diabetic individuals, IRAK-1 gene expression correlated significantly only with MyD88 expression (r = 0.52, *P* = 0.0003) and had no association with TLR2 (r = 0.19, *P* = 0.20) and TLR4 (r = −0.06, *P* = 0.60) expression (Fig. 6d–f).

## Discussion

IRAK-1 is a critical adapter protein of the TLR/IL-1R/MyD88 signaling pathway and obesity-associated changes in its expression remain unclear. In the present study, we have identified IRAK-1 as a tissue marker of meta-inflammation in obesity by using gene expression analysis in the subcutaneous adipose tissue. IRAK-1 is an IL-1R-associated serine/threonine kinase and MyD88 is a proximal adapter protein that facilitates IRAK4-mediated phosphorylation of IRAK-1. As a key component of the TLR/IL-1R/MyD88 signaling pathway, IRAK-1 is an adapter protein and kinase that regulates the formation of the TRAF-6/TAK-1/TAB-1/2 macromolecular signaling complex. Later on, through a series of sequential events, the dissociation of hyperphosphorylated IRAK-1 from the macromolecular complex results in phosphorylation, ubiquitination, and degradation of IκB-α, leading eventually to the activation and nuclear translocation of p65 NF-κB complex [14, 15]. The previous studies reported roles of MyD88 and/or IRAK adapter proteins in the innate immunity [9, 14–19], inflammation and cancer metastasis [20–23]. In the present study, we show that IRAK-1 gene expression was significantly elevated in the adipose tissues of obese and overweight non-diabetic individuals as compared with lean subjects and the changes correlated with clinical hallmarks of obesity including BMI and fat % (Fig. 1a–c). However, in the adipose tissue samples from diabetic individuals, IRAK-1 gene expression was comparably upregulated in both overweight and obese individuals and the difference between obese and lean subjects was found to be significant; whereas IRAK-1 correlated non-significantly with BMI and fat % (Fig. 1d–f). In T2D, redistribution changes of lipolysis occur in different adipose regions, leading to fatty acid mobilization in the favor of visceral fat depot. The metabolic perturbations also impair TLR signaling pathways, such as those involving MyD88 and IRAK-1. The lack of correlation between IRAK-1 and BMI or fat % in diabetic patients may be because this group is more heterogeneous with regard to immune-metabolic changes (as mentioned above), comorbid factors, and T2D therapeutic interventions. The data also show that IRAK-1 protein expression was remarkably elevated in the obese adipose tissues of both non-diabetic and diabetic individuals (Fig. 2a–i).

The white adipose tissue is a site for excessive energy storage and is also an active endocrine organ that secretes adipokines [24]. We sought to determine how the changes in the adipose tissue expression of IRAK-1 related with local expression of typical proinflammatory cytokines. To this end, we found that in non-diabetic individuals, the transcript levels of TNF-α, IL-6, and IL-18 were significantly upregulated in the adipose tissues from obese and overweight individuals as compared with lean counterparts and this inflammatory profile of the obese adipose tissue correlated directly with the IRAK-1 gene expression (Fig. 3a–c). However, in diabetic patients, only the TNF-α mRNA expression correlated significantly with IRAK-1 gene expression in the adipose tissue (Fig. 3d–f). Thus, TNF-α correlated with IRAK-1 gene expression in both non-diabetic and diabetic individuals. TNF-α and IL-6 are proinflammatory cytokines expressed by both adipocytes and macrophages [25, 26]. IL-18, also known as IFN-γ-inducing factor, is a proinflammatory cytokine expressed by macrophages, adipocytes and other cell types and it has been associated with several inflammatory conditions [27, 28]. Our data showing elevated IL-18 gene expression in the adipose tissue of obese and overweight individuals are concordant with previous studies reporting the increased IL-18 mRNA expression in obese adipose tissue [29] or in activated adipocytes [30]. Our data represent obesity/T2D-related inflammatory changes in the subcutaneous adipose tissue which is easily accessible by transcutaneous biopsy for clinical studies. Of note, a previous study reported that both the subcutaneous and visceral depots represented the inflammatory changes related with insulin resistance in human obesity [31]. However, others showed that the changes including systemic inflammation, atherosclerosis, and metabolic syndrome were more pronounced in the visceral as compared with subcutaneous adipose tissue [32–34]. In any case, the lack of visceral adipose tissue data may be regarded as a limitation of this study which is listed as one of caveats at the end of discussion.

Apart from obesity- or T2D-related changes in adipocytes, altered composition of the stromal vascular cell fraction can also modulate the inflammatory state of adipose tissue. The stromal cell fraction comprises of different cell types including preadipocytes, fibroblasts, endothelial cells, progenitor cells, nerve cells, and importantly, immune effector cells [35]. Macrophages are among the most abundant immune cells that are found in the stromal cell fraction and have been reported to accumulate in the expanded adipose tissue mass in obesity [36–38]. In the advanced stages of obesity, different types of stromal immune cells such as T lymphocytes, neutrophils, and monocytes/macrophages infiltrate into the adipose tissue and trigger the inflammatory changes via a crosstalk with parenchymal adipocytes. The adipose tissue infiltration of monocytes/macrophages can be assessed by detecting expression of multiple selection markers. In line with this, as expected, we found that the elevated IRAK-1 gene expression in non-diabetics correlated positively with CD68, CD11c, and CD163 macrophage marker expression in the obese adipose tissues (Fig. 4a–c); whereas IRAK-1 correlated positively/significantly only with CD163 in diabetic patients (Fig. 4d–f). CD68 is a 110 kDa transmembrane glycoprotein that is highly expressed on human monocytes and tissue macrophages; however, it is also expressed by basophils, neutrophils, and lymphoblasts [39]. The CD68 protein is a member of the lysosome-associated membrane glycoprotein (LAMP) family and scavenger receptor family. Rapid recirculation of the CD68 from endosomal/lysosomal compartments to the plasma membrane allows macrophage movements over selectin-expressing cells or substrates which plays a key role in homing and phagocytic activities of the tissue macrophages. CD11c, also known as integrin-αx (ITGAX) or CR4, is a 145–150 kDa type-I transmembrane protein that is highly expressed on dendritic cells, and is also expressed by monocytes/macrophages, granulocytes, natural killer (NK) cells, and T/B lymphocytes. It plays a role in cell adhesion and is considered a monocyte/macrophage activation marker [40]. CD163 is a 130 kDa scavenger receptor which is exclusively expressed by monocytes/macrophages. It is an acute phase-regulated receptor and is involved in clearance and endocytosis of hemoglobin-hapatoglobin complexes by macrophages [41]. After its shedding during inflammatory conditions such as T2D, hepatic cirrhosis, rheumatoid arthritis, Hodgkin lymphoma, and sepsis; the soluble form (sCD163) can play an anti-inflammatory role and is considered to be a diagnostic marker for macrophage activation [42]. The positive correlation between increased IRAK-1 gene expression and these markers in obesity indicates the concordance of the activation of TLR/IL-1R/MyD88 pathway and macrophage recruitment or colonization in the adipose tissue to induce or sustain inflammation. Similarly, a previous study reported an interplay among obesity, inflammation, and macrophage chemotaxis in breast adipose tissue [43]. Overall, our data point to a concordance of IRAK-1 gene expression with the inflammatory mediators and macrophage markers, implying that the increased IRAK-1 gene expression in the obese adipose tissue represents a global inflammatory change in this compartment. The link between IRAK-1 expression and obesity-associated meta-inflammation is also supported by the relationship of IRAK-1 or IRAK-2 with other inflammatory conditions such as lung cancer [44], systemic lupus erythematosus [45], Vogt-Koyanagi-Harada (VKH) disease [46], and Alzheimer’s disease [47].

We further found that IRAK-1 gene expression in non-diabetics correlated with the plasma levels of CCL-5, CRP, adiponectin, and triglycerides (Fig. 5a–d); while in diabetic patients, IRAK-1 correlated significantly only with plasma triglycerides (Fig. 5d–h). CCL-5 or RANTES is a C–C motif chemokine which acts as a key regulator of leukocyte migration into the sites of inflammation [48]. CRP is a hepatokine and a component of the acute phase reaction. Elevated CRP circulatory levels were found in inflammatory conditions such as Crohn’s disease, inflammatory bowel disease, ulcerative colitis, infections, necrosis, malignancy, sepsis, and severe trauma [49–51]. Our data showing higher circulatory levels of CCL-5 and CRP in non-diabetics suggest the presence of systemic inflammation in obese individuals as compared with overweight and lean subjects. CCL-5 and CRP expression may be cooperatively linked as a previous study reported that CRP induced cellular activation and production of CCL-5 in the renal tubular epithelial cells in a dose-dependent manner [52]. Adiponectin is a protein hormone secreted by adipose tissue and it is involved in glucose regulation and fatty acid oxidation. The circulatory levels of adiponectin were reported to correlate inversely with body fat percentage [53]. IRAK-1 gene expression that showed a positive relationship with fat % in our study, as expected, was found to correlate negatively with the plasma adiponectin levels. On the other hand, IRAK-1 gene expression correlated positively with plasma triglycerides. Notably, a significant correlation between obesity and plasma triglyceridemia has been previously reported [54]. However, not all obese individuals are found to be hypertriglyceridemic which implies that triglyceride levels may or may not be related to the circulating lipoproteins. We found that plasma triglycerides correlated inversely with IRAK-1 gene expression in the adipose tissue of diabetic patients. Increased triglyceride levels in obesity were reported as a risk factor for cardiovascular disease, independently of the high-density lipoprotein cholesterol levels [55] and hypertriglyceridemia with low high-density lipoprotein cholesterol was described as a key feature of the metabolic syndrome in obese individuals [56].

TLRs are important regulators of the innate immune system that recognize pathogen- or danger-associated molecular patterns and TLR intracellular signaling is mediated via adapter proteins such as MyD88 and TRIF. Our data show the upregulated gene expression of TLR2 and MyD88 in the adipose tissue of obese individuals. The IRAK-1 gene expression correlated positively/significantly with both TLR2 and MyD88 in non-diabetics while it correlated with MyD88 in diabetic patients (Fig. 6a–f). TLR signaling activates transcription factors that increase expression of various inflammatory markers [57]. Increased TLRs expression has been reported in other inflammatory conditions as well, such as asthma, inflammatory bowel disease, rheumatoid arthritis, and type-1 diabetes [58–61]. The hallmark of diabetes is hyperglycemia and several studies showed that hyperglycemia could cause increased TLR2/4 expression and activity in different cell types [62–64]. We found the increased TLR2/MyD88 gene expression in the adipose tissue of obese/diabetic individuals which correlated with IRAK-1 while no significant changes were observed in the TLR4 expression. The free fatty acids found in the circulation of obese/diabetic individuals may act as TLR2/4 ligands to promote inflammatory signaling [65, 66]. Thus, it may be speculated that hyperglycemia and free fatty acids may play a significant role in inducing and activating inflammatory signaling via TLR2/4 pathways. Notably, high fat diet fed TLR2^−/−^ mice were found to be protected from insulin resistance and β-cell dysfunction [67]. However, depending on type of ligand binding, TLR2 may have both pro- and anti-inflammatory effects as TLR2 ligands such as zymosan and lectin-1 resulted in the expression of anti-inflammatory TGF-β [68, 69]. Also, zymosan treatment was reported to decrease hyperglycemia and increase regulatory T cell numbers in nonobese diabetic severe combined immunodeficiency mice [69]. Previously, we reported the increased TLR2/4 and MyD88/IRAK-1 expression in the peripheral blood mononuclear cells of type-2 diabetic individuals but we could not detect IRAK-1 expression in the adipose tissue of this patient cohort by using immunohistochemistry [12]. In the present study, we therefore analyzed the IRAK-1 expression at both gene and protein expression levels by using quantitative real time RT-PCR and immunohistochemistry/confocal microscopy, respectively. Notably, in the present study, a different anti-IRAK-1 antibody (ab62700, Abcam) was used for immunohistochemical staining than the antibody (ProSci Incorporated) used in previous study. The reactivity of anti-IRAK1 antibodies may vary in immune cells and adipose tissue due to either different IRAK1 isoforms present or different target epitopes recognized by different antibodies.

The present data suggest that the increased IRAK-1 expression may be an additional biomarker for the adipose tissue inflammatory state in obesity. Nonetheless, caution is warranted while interpreting these results as our study is limited by a few caveats as follows: (1) the data represent obesity/T2D-associated changes in the subcutaneous adipose tissue while changes in the visceral adipose tissue remain unclear; (2) adipose tissue samples were not fractionated into adipocytes, macrophages and stromal cells for total RNA isolation and thus the qPCR data alone may not be able to assess target gene expression in a particular cell type; (3) it remains to be seen whether the expression of other TLRs, such as TLR5, which is expressed on both immune and non-immune cells and is a receptor for flagellin associates with IRAK-1 expression in obesity and/or T2D; and (4) mechanistically, it would also be interesting to see whether the expression levels of regulatory microRNAs, such as miR-146a which is a known negative feedback regulator of IRAK-1/TRAF-6 and miR-21 which is a suppressor of TLR2 signaling, are deregulated in obesity or T2D. Therefore, further studies on these aspects will be required to validate and extend these preliminary findings.

## Conclusions

Taken together, our data show that in the subcutaneous adipose tissue, IRAK-1 expression was significantly upregulated in obese and overweight individuals as compared with lean subjects. Based on consensus with both circulatory and local inflammatory signatures, IRAK-1 expression may be regarded as an additional tissue marker for meta-inflammation in obesity. The study has clinical significance as the approaches intending IRAK-1 suppression may improve metabolic complications in obesity or T2D.

## Additional files

Additional file 1:
**Figure S1.** Comparative IRAK-1 protein expression in obese, overweight, and lean individuals. The protein expression of IRAK-1 in non-diabetic obese, overweight, and lean adipose tissue samples, 5 each, was determined by using immunohistochemistry as described in Patients and Methods. Analysis of the IRAK-1 intensity determined by using Aperio positive pixel count algorithm software (version 9) revealed a significantly higher expression in obese individuals as compared with lean subjects (*P*=0.0317). Additional file 2:
**Figure S2.** Comparison of IRAK-1 gene and protein expression in the adipose tissue. The gene and protein expression of IRAK-1 in non-diabetic obese, overweight, and lean adipose tissue samples, 5 each, were determined by using real-time RT-PCR and immunohistochemistry, respectively, as described in Patients and Methods. The relative mRNA expression was measured as fold expression over average of control gene expression taken as 1. The protein expression was measured as intensity which was calculated by using Aperio positive pixel count algorithm software (version 9).

## Abbreviations

IRAKs: interleukin (IL)-1 receptor-associated kinases
TLRs: toll-like receptors
PAMPs: pathogen-associated molecular patterns
MyD88: myeloid differentiation factor 88
TRAF: TNF receptor-associated factor
TAK: TGFβ-activated kinase
IKKα/β: inhibitor of nuclear factor kappa-B kinase subunits alpha/beta
NF-κB: nuclear factor kappa-B
MAPK: mitogen-activated protein kinase
MKK: MAPK kinase
JNK: c-Jun N-terminal kinase
T2D: Type-2 diabetes
BMI: body mass index
Fat %: fat percentage
PBS: phosphate buffered saline
OCT: optimal cutting temperature
RT-PCR: reverse-transcription polymerase chain reaction
MGB: minor groove binder
FAM: fluorescein amidite
NFQ: non-fluorescent quencher
UDG: uracil DNA glycosylase
DAB: 3,3ʹ-diaminobenzidine
DPX: dibutyl phthalate xylene
DAPI: 4′,6-diamidino-2-phenylindole
HRP: horse radish peroxidase
TNF: tumor necrosis factor
RANTES: regulated on activation, normal T cell expressed and secreted
CRP: C-reactive protein
VKH: Vogt-Koyanagi-Harada
LAMP: lysosome-associated membrane glycoprotein
ITGAX: integrin-alpha х
NK: natural killer
