# Supplementary figures and images for: Increased expression of the interleukin-1 receptor-associated kinase (IRAK)-1 is associated with adipose tissue inflammatory state in obesity
Source: Diabetol Metab Syndr. 2015 Aug 27;7:71. doi: 10.1186/s13098-015-0067-7 (PMC4549832; doi:10.1186/s13098-015-0067-7)

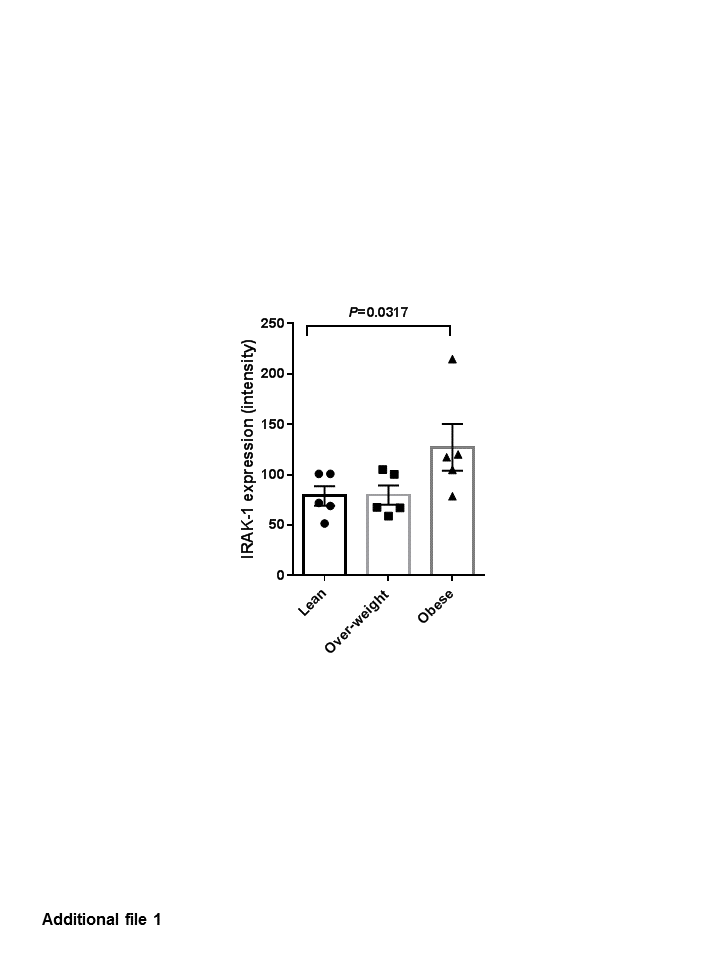

Supplement: Additional file 1: — Figure S1. Comparative IRAK-1 protein expression in obese, overweight, and lean individuals. The protein expression of IRAK-1 in non-diabetic obese, overweight, and lean adipose tissue samples, 5 each, was determined by using immunohistochemistry as described in Patients and Methods. Analysis of the IRAK-1 intensity determined by using Aperio positive pixel count algorithm software (version 9) revealed a significantly higher expression in obese individuals as compared with lean subjects (P=0.0317). [file 13098_2015_67_MOESM1_ESM.png]

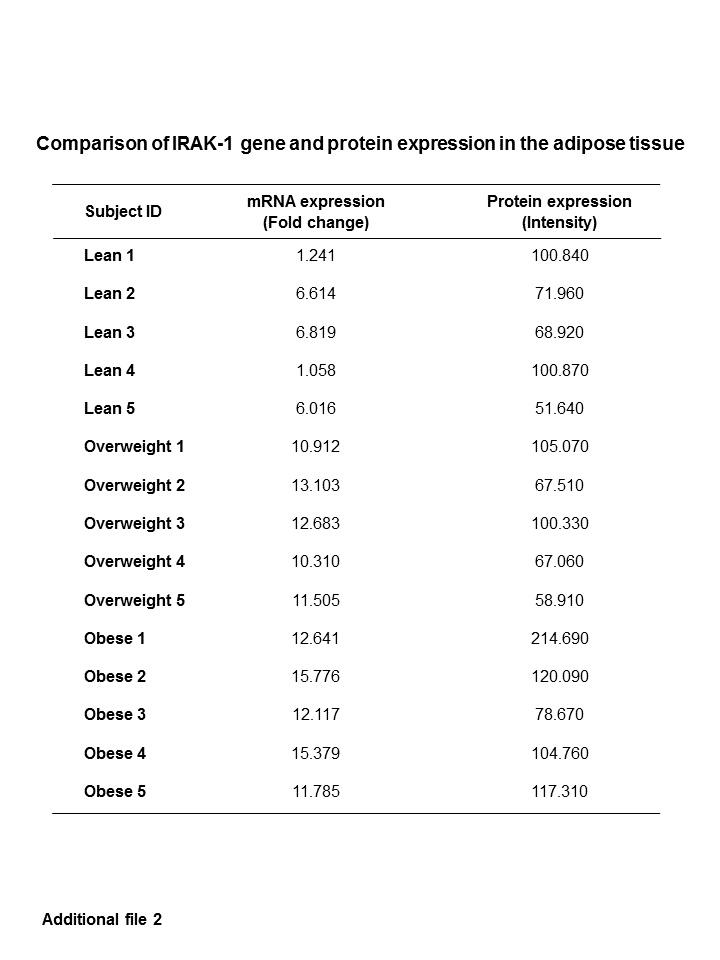

Supplement: Additional file 2: — Figure S2. Comparison of IRAK-1 gene and protein expression in the adipose tissue. The gene and protein expression of IRAK-1 in non-diabetic obese, overweight, and lean adipose tissue samples, 5 each, were determined by using real-time RT-PCR and immunohistochemistry, respectively, as described in Patients and Methods. The relative mRNA expression was measured as fold expression over average of control gene expression taken as 1. The protein expression was measured as intensity which was calculated by using Aperio positive pixel count algorithm software (version 9). [file 13098_2015_67_MOESM2_ESM.png]
